# Preliminary Screening on Antibacterial Crude Secondary Metabolites Extracted from Bacterial Symbionts and Identification of Functional Bioactive Compounds by FTIR, HPLC and Gas Chromatography–Mass Spectrometry

**DOI:** 10.3390/molecules29122914

**Published:** 2024-06-19

**Authors:** Gobinath Chandrakasan, Juan Fernando García-Trejo, Ana Angelica Feregrino-Pérez, Humberto Aguirre-Becerra, Enrique Rico García, María Isabel Nieto-Ramírez

**Affiliations:** División de Estudios de Posgrado, Facultad de Ingeniería, Universidad Autónoma de Querétaro, Santiago de Querétaro 76010, Querétaro, Mexico; feregrino.angge@hotmail.com (A.A.F.-P.); humbertoagbe@hotmail.com (H.A.-B.); ricog@uaq.mx (E.R.G.); isabel.nieto@uaq.mx (M.I.N.-R.)

**Keywords:** entomopathogenic nematodes, symbiotic bacteria, PCR, antibacterial activity, HPLC, GC–MS

## Abstract

Secondary metabolites, bioactive compounds produced by living organisms, can unveil symbiotic relationships in nature. In this study, soilborne entomopathogenic nematodes associated with symbiotic bacteria (*Xenorhabdus stockiae* and *Photorhabdus luminescens*) were extracted from solvent supernatant containing secondary metabolites, demonstrating significant inhibitory effects against *E. coli*, *S. aureus*, *B. subtilus*, *P. mirabilis*, *E. faecalis*, and *P. stutzeri*. The characterization of these secondary metabolites by Fourier transforms infrared spectroscopy revealed amine groups of proteins, hydroxyl and carboxyl groups of polyphenols, hydroxyl groups of polysaccharides, and carboxyl groups of organic acids. Furthermore, the obtained crude extracts were analyzed by high-performance liquid chromatography for the basic identification of potential bioactive peptides. Gas chromatography–mass spectrometry analysis of ethyl acetate extracts from *Xenorhabdus stockiae* identified major compounds including nonanoic acid derivatives, proline, paromycin, octodecanal derivatives, trioxa-5-aza-1-silabicyclo, 4-octadecenal, methyl ester, oleic acid, and 1,2-benzenedicarboxylicacid. Additional extraction from *Photorhabdus luminescens* yielded functional compounds such as indole-3-acetic acid, phthalic acid, 1-tetradecanol, nemorosonol, 1-eicosanol, and unsaturated fatty acids. These findings support the potential development of novel natural antimicrobial agents for future pathogen suppression.

## 1. Introduction

Natural compounds derived from living organisms exhibit remarkable chemical diversity and hold immense promise for discovering new compounds with novel modes of action. Many organisms found in nature, including fungi, plants, and bacteria, produce antimicrobial compounds as secondary metabolites to compete with other organisms [1,2,3]. One of the biocontrol sources of *Photorhabdus* and *Xenorhabdus* genera demonstrates novel antimicrobial peptides. These bacteria encode several putative biosynthetic pathways for natural product biosynthesis and play crucial ecological roles [4,5]. Notably, *Photorhabdus* and *Xenorhabdus* symbiotic bacteria are associated with entomopathogenic nematodes (EPNs), which are obligate and lethal insect parasites [6,7]. Once nematodes enter an insect, they release their symbiotic bacteria into the insect hemolymph; within 2 to 3 days, the insect is killed by bacterial toxins and enzymes [8,9].

Effective methods are essential for harnessing bacterial extracellular products for pest control and drug discovery applications. These symbiotic bacteria inhabit the digestive tract of infective juveniles during their primary phase but will transition into a secondary phase under in vitro conditions. Moreover, biocontrol mechanisms employed by bacterial symbionts result in the host’s demise and subsequent proliferation. To sustain optimal conditions for nematode reproduction, it is necessary to provide nutrients and antimicrobial substances that impede the growth of a wide range of microorganisms within and outside the cadaver. Additionally, there is potential for the production of supplementary bioactive compounds, such as uncharacterized secondary metabolites, by these symbionts. The biosynthesis of antimicrobial and nematicidal metabolites strongly suggests their role in preventing the consumption of the insect cadaver by competing organisms. These bacteria can be cultured as free-living organisms under controlled laboratory conditions. Within unique temperature, pH, and humidity parameters, they secrete a diverse array of virulent factors, including high-molecular-weight toxin complexes, lipopolysaccharides, proteases, and various antibiotics, all of which can be evaluated in the culture media.

Most species of *Xenorhabdus* and *Photorhabdus* produce multiple groups of active secondary metabolites, with *Xenorhabdus* exhibiting greater diversity compared to *Photorhabdus* [10,11]. These metabolites play significant roles in various bioactivities relevant to pharmaceutical and agricultural fields [12], encompassing antibiotic, antimycotic, insecticidal, nematicidal, antiulcer, antineoplastic, and antiviral properties [13]. Consequently, nematode symbiotic bacteria represent an original source for potential biomedical applications [14]. These symbiotic bacteria undergo complex life cycles, and their insect hosts succumb rapidly to insecticidal bacterial toxins [14]. Extensive studies are necessary to deepen our understanding of symbiotic bacteria and their secretions. Various types of antibiotics against bacteria and fungi are synthesized and secreted within bacterial cultures of these symbiotic bacteria [15]. Against this backdrop, the present moment presents an opportune time for the exploration and production of bacterial metabolites for the control of bacterial pathogens. In the study described here, the extraction of crude metabolites from *Xenorhabdus* and *Photorhabdus* is undertaken with the aim of developing applications in antimicrobial usage. These findings will contribute to a better understanding of bacterial symbiont metabolites, potentially yielding novel compounds beneficial for agricultural and pharmaceutical purposes.

## 2. Results

### 2.1. Extraction of Symbiotic Bacteria

**Figure 1:**
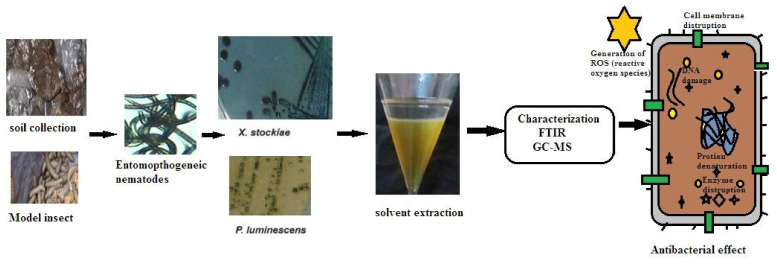
The general view on natural antibacterial crude secondary metabolites extracted from bacterial symbionts and identification of functional bioactive compounds by FTIR and gas chromatography–mass spectrometry.

A total of 10 nematodes associated with symbiotic bacteria (beneficial for soil) were isolated from various agriculture sites in Amazcala, Queretaro, Mexico (Table 1). Out of the ten nematodes recovered, two potential symbiotic bacteria, strains 05 and 10, were found to exhibit significant antibacterial activity against pathogens. Strains 5 and 10 were selected for bacterial isolation and molecular characterization. The symbiotic bacteria were isolated and identified through direct and indirect confirmation methods. At the primary level, the identification of symbiotic bacterial colonies was based on the absorption of dyes such as bromothymol blue and triphenyl tetrazolium from NBTA plates. Colonies appeared as blue-green, blue, or brownish, depending on species variation. Based on our observations of colony morphology, the two strains belonged to the genera *Xenorhabdus* spp. for *Steinernema* spp. and genera *Photorhabdus* spp. for *Heterorhabditis* spp. Figure 1 illustrates the general view on natural antibacterial crude secondary metabolites extracted from bacterial symbionts and the identification of bioactive functional compounds using high-performance liquid chromatography (HPLC) and gas chromatography–mass spectrometry (GC–MS).

### 2.2. Bacterial Identification by 16S rRNA

**Figure 2:**
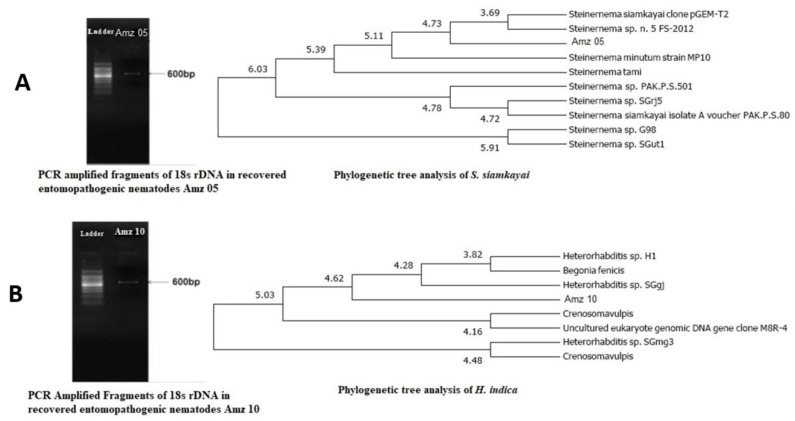
(**A**) Recovered soil entomopathogenic nematodes’ molecular characterization (strains 05 and 10); (**B**) extracted symbiotic bacteria molecular characterization (strains 05 and 10). BLAST search indicated more than 98% similarity between the sequences of the PCR product and the recovered sample. The sequence of the phylogenetic tree was constructed by Untitled Clustal W (Slow/Accurate, IUB).

For further authentication, the isolated symbiotic bacteria were identified at the molecular level via 16S rRNA gene sequencing. The symbiont was amplified using PCR primers representing regions of the 16s rRNA conserved in bacteria. Each strain produced a single band of approximately 1450 bp. The 16s rRNA strain sequences (strains 05 and 10) were determined and showed 98–99% similarity with that of *X. stockiae* and *P. luminescens*, respectively. Figure 2 displays (A) the molecular characterization of the recovered soil entomopathogenic nematodes (strains 05 and 10) and (B) the molecular characterization of the extracted symbiotic bacteria (strains 05 and 10).

### 2.3. Functional Group Analysis by FTIR

**Figure 3:**
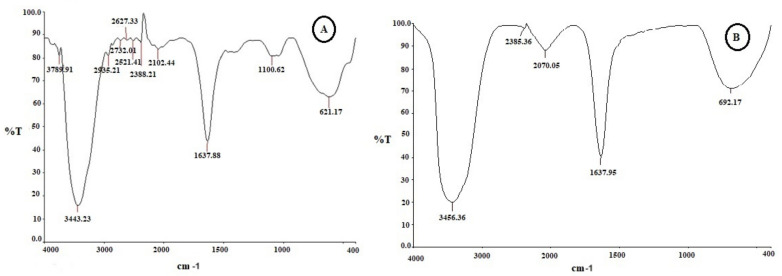
The FTIR analysis of bacterial crude compounds of (**A**) *X. stockiae* and (**B**) *P. luminescens*.

The FTIR spectrum recorded for the cell-free supernatant of *X. stockiae* displayed peaks at 3443, 1637, 1100, and 621 cm^−1^. Figure 3 features the FTIR analysis of bacterial crude compounds of (A) *X. stockiae* and (B) *P. luminescens*. Table 2 presents the FTIR spectrum peaks of the *X. stockiae* crude compound in its possible assigned functional groups. In the resultant FTIR characterization spectra of bacterial culture extracts, major shifts were observed from 3436 cm^−1^ to 3443 cm^−1^. Table 3 displays the FTIR spectrum peaks of the *P. luminescens* crude compound in its possible assigned functional groups.

### 2.4. Purification of Secondary Functional Compound Using HPLC and GC–MS

The active fractions were then pooled together and purified through column chromatography, and further purified HPLC fractions were obtained. Each fraction was investigated in terms of antibacterial activity. Based on our previous studies, we screened potential antibacterial activity, namely against *X. stockiae* for fraction 5 and *P. luminescens* for fraction 4. These two fractions were tested against *E. coli*, *S. aureus*, *B. subtilus*, *P. mirabilis*, *E. faecalis*, and *P. stutzeri.* Table 4 describes the eight potential HPLC fractions obtained from bacterial crude extracts of *X. stockiae*. Table 5. describes the eight potential HPLC fractions obtained from bacterial crude extracts of *P. luminescens.* Afterward, we chose the HPLC fractions with different degrees of purification, with relevant antimicrobial activity, which were subjected to LC-MS analysis to determine the molecular masses of the metabolites with antimicrobial activity produced by *X. stockiae* and *P. luminescens*.

**Figure 4:**
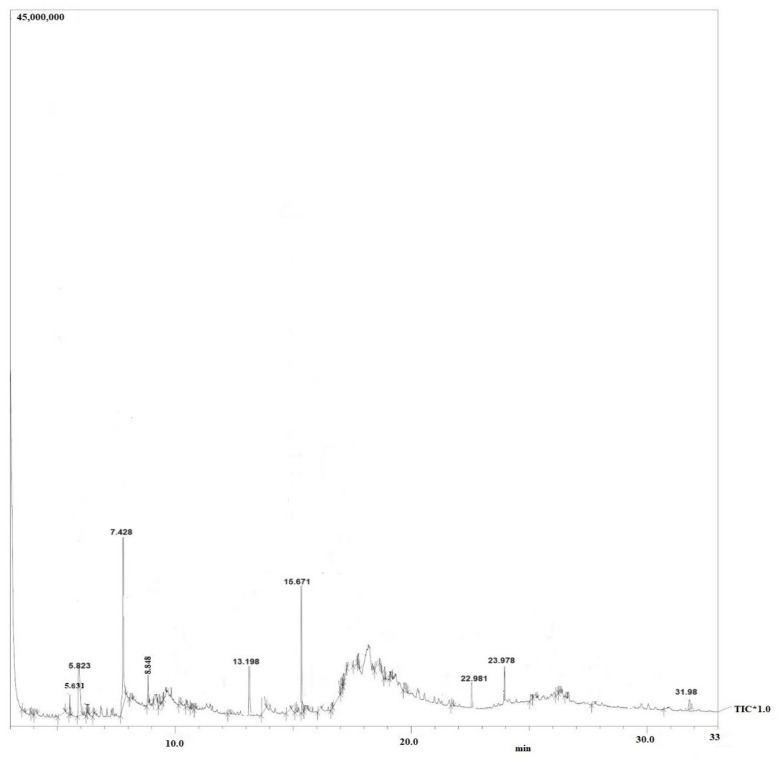
GC–MS chromatogram of *X. stockiae*.

**Figure 5:**
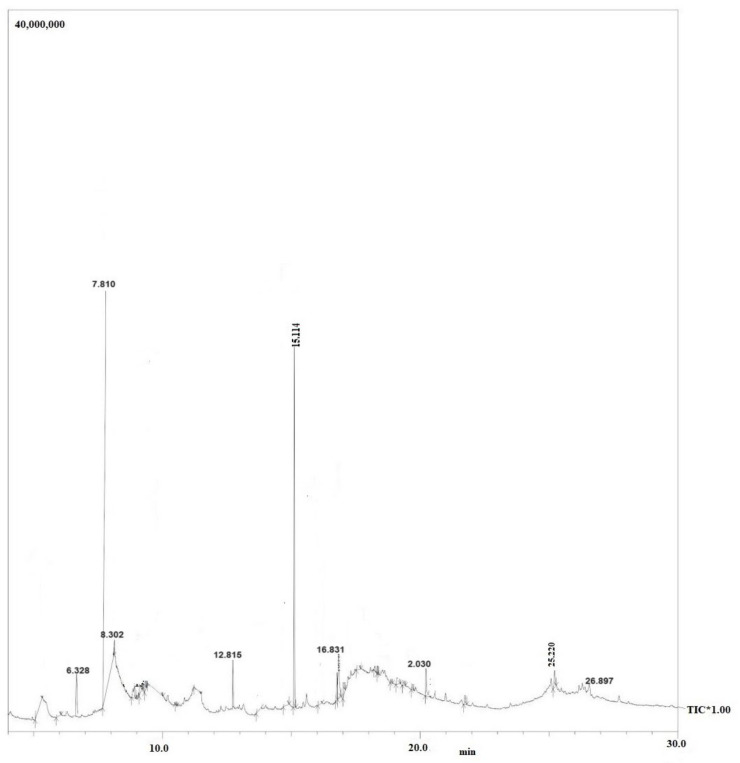
GC–MS chromatogram of *P. luminescens*.

Moreover, purified secondary metabolites were analyzed using GC–MS to gain a basic understanding of the metabolites present and compare the chemical profiles of each peak. Figure 4 illustrates the GC–MS chromatogram of *X. stockiae*, while Figure 5 shows the GC–MS chromatogram of *P. luminescens*. Table 6 summarizes the fragments and retention times for significant compounds identified in the *X. stockiae* strain from ethyl acetate crude extract. GC peaks corresponded to various compounds with reported biological activity, including nonanoic acid derivatives, paromycin, pyrrolidinone, octodecanal derivatives, trioxa-5-aza-1-silabicyclo, 4-octadecenal, methyl ester, oleic acid, and 1,2-benzenedicarboxylicacid. Table 7 presents a summary of the fragments and retention times for significant compounds identified in the *P. luminescens* strain from ethyl acetate crude extract. Additional extraction from *P. luminescens* yielded functional compounds such as indole-3-acetic acid, piperidinol derivatives, phthalic acid, 1-tetradecanol, nemorosonol, 1-eicosanol, and unsaturated fatty acids.

### 2.5. Antibacterial Activities of Symbiotic Bacteria

**Figure 6:**
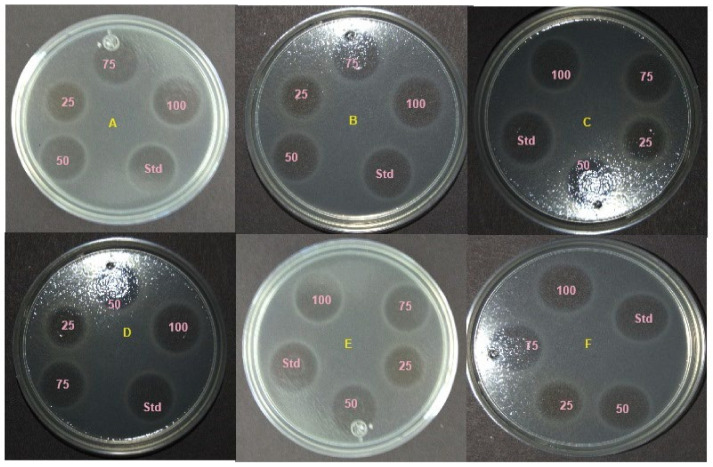

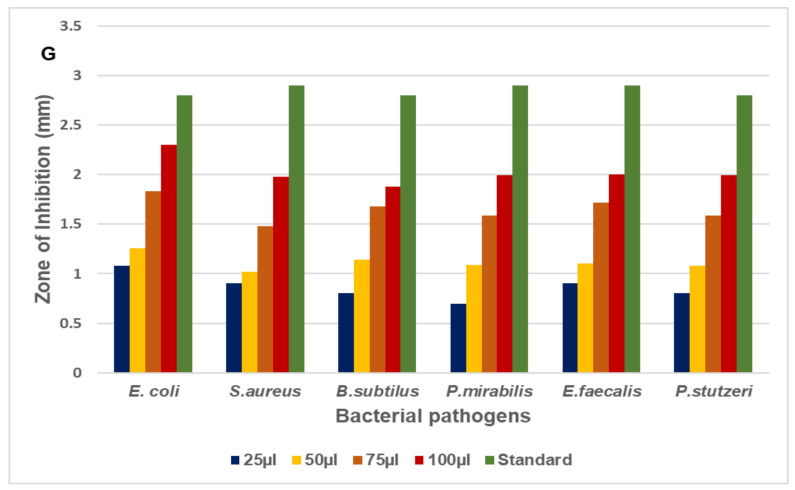
Screening of antibacterial effect on *X. stockiae* fractionated compounds using HPLC analysis at different concentrations (25 µL, 50 µL, 75 µL, 100 µL, and standard antibiotics). Bacterial pathogens: (**A**) *E. coli*, (**B**) *S. aureus*, (**C**) *B. subtilus*, (**D**) *P. mirabilis*, (**E**) *E. faecalis*, and (**F**) *P. stutzeri*; (**G**) bar graph indicating ZOI of bacterial pathogens with bioactive substances of *X. stockiae*.

**Figure 7:**
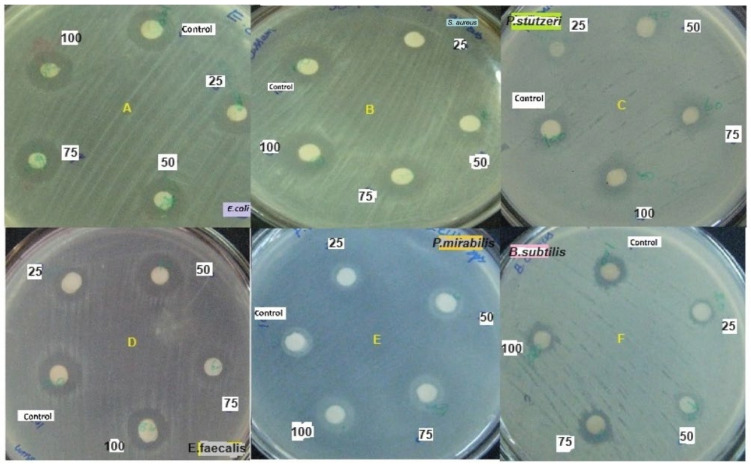

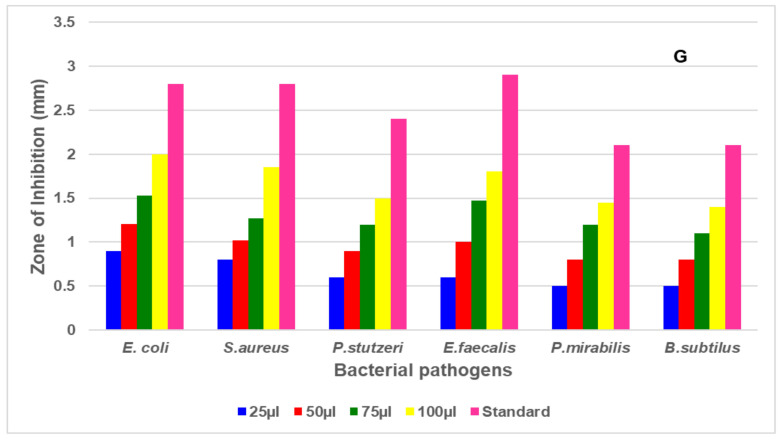
Screening of antibacterial effect on *P. luminescens* fractionated compounds using HPLC analysis at different concentrations (25 µL, 50 µL, 75 µL, 100 µL, and standard antibiotics). Bacterial pathogens: (**A**) *E. coli*, (**B**) *S. aureus*, (**C**) *P. stutzeri*, (**D**) *E. faecalis*, (**E**) *P. mirabilis*, and (**F**) *B. subtilus*; (**G**) bar graph indicating ZOI of bacterial pathogens with bioactive substances of *P. luminescens*.

Our study revealed that the ethyl acetate extract of the crude compound from *X. stockiae* and *P. luminescens* could inhibit up to six strains of pathogenic bacteria, namely *E. coli*, *S. aureus*, *B. subtilus*, *P. mirabilis*, *E. faecalis*, and *P. stutzeri*. The highest ZOI was found for *E. coli*, *E. faecalis*, and *S. aureus*. Moderate levels of ZOI were observed for *B. subtilus*, *P. mirabilis*, and *P. stutzeri*. Figure 6 illustrates the screening results regarding the antibacterial effect on *X. stockiae* fractionated compounds using HPLC analysis at different concentrations (25 µL, 50 µL, 75 µL, 100 µL, and standard antibiotics). Bacterial pathogens were (A) *E. coli*, (B) *S. aureus*, (C) *B. subtilus*, (D) *P. mirabilis*, (E) *E. faecalis*, and (F) *P. stutzeri*, and (G) a bar graph indicating the ZOI of bacterial pathogens with bioactive substances of *X. stockiae* is also shown. Figure 7 demonstrates the screening results regarding the antibacterial effect on *P. luminescens* fractionated compounds using HPLC analysis at different concentrations (25 µL, 50 µL, 75 µL, 100 µL, and standard antibiotics). Bacterial pathogens were (A) *E. coli*, (B) *S. aureus*, (C) *P. stutzeri*, (D) *E. faecalis*, (E) *P. mirabilis*, and (F) *B. subtilus*, and (G) a bar graph indicating the ZOI of bacterial pathogens with bioactive substances of *P. luminescens* is also shown.

## 3. Discussion

These symbiotic bacteria are closely related to the family Enterobacteriaceae. The phylogeny of these organisms is well defined, as they are clearly placed in the gamma group of proteobacteria [33]. Each isolate was associated with a distinct bacterial genus belonging to different *X. stockiae* and *P. luminescens*. Only a few strains of the symbiotic bacteria have been described and studied in detail, and their molecular biology has been elucidated by previous research [34]. The primary phase is the major antibiotic-producing phase, whereas the second phase produces fewer antibiotic molecules. Bacteria in this genus have also been shown to be rich in natural products such as insecticidal toxins, inhibitors of the insect immune system, and a variety of antibiotics that facilitate nematode infection [35]. These metabolites have been utilized in biological pesticides and therapeutic agents for many decades. In our study, we conducted the screening of antimicrobial crude secondary metabolites extracted from bacterial strains for further characterization.

The broad-spectrum peak at 3443 cm^−1^ corresponds to the strong stretching vibrations of the hydroxyl functional group [36]. The bands at 1100 and 1637 cm^−1^ correspond to the stretching vibrations of amide N-H (ANAH) and carbonyl (CAOA) groups in amide linkages (amide I and amide II) of the protein present in bacterial supernatant [37]. The small peak at 621 cm^−1^ is characteristic of carbonyl stretching vibrations in the amide II functional group [38]. The observed FTIR spectrum results confirm the presence of the hydroxyl functional group as well as a slight shift in all peak positions and absorption bands. These features may also contribute to antimicrobial and anticancer properties. *Xenorhabdus* spp. have been reported to produce antimicrobial activity; indole compounds identified in culture broths of several *Xenorhabdus* spp. showed antibacterial and antifungal activity [39,40].

Fabclavines are polyketide-derived compounds produced by *Xenorhabdus* bacteria. In GC–MS outcomes, the proline functional group is involved in the late steps of antimicrobial peptide production such as fabclavines [41] [see the Supplementary Materials (Figures S1–S10) following some important functional bioactive compounds (ten) located in the crude extract on *X. stockiae* and *P. luminescens* using Gas chromatography-mass spectrometry with main constituents through NISIT Library [1,2- Benzenedicarboxylic acid, Acetic acid, Hy-droxybenzoic acid, Octodecenol, Nonanoic acid, Octodecanic acid, Oleic acid methyl ester, Phthalic acid, Piperidenyl, Tetradecanic acid and Linoleic acid]]. These compounds have been shown to possess antimicrobial activity against a variety of bacteria, including Gram-positive and Gram-negative pathogens. Xenocoumacins exert their antimicrobial effects by inhibiting bacterial RNA polymerase, leading to the suppression of bacterial growth and viability. Additionally, GC–MS results showed that *P. luminescens* produced secondary metabolites of 1-tetradecanol compounds. Recently, these compounds have been extracted from nematode symbiotic bacteria and evaluated against insect pathogens (*Pieris rapae* and *Pentodon algerinus*) [42]. Another important and previously evaluated secondary metabolite compound, 1, 2, benzenedicarboxylic acid, represents a potential antibacterial agent. Similarly, different kinds of *Xenorhabdus* strains produce many bioactive compounds with antibacterial, antifungal, and cytotoxicity properties [43]. Furthermore, octadecanoic acid and methyl ester extracted from *Photorhabdus* bacteria have potential antimicrobial, anti-inflammatory, antioxidant, and antibacterial activities based on earlier studies [44,45]. Photorhabdus bacteria produce phenazines, nitrogen-containing heterocyclic compounds with broad-spectrum antimicrobial activity. Phenazines act by generating reactive oxygen species within bacterial cells, causing oxidative damage to proteins, lipids, and DNA. This oxidative stress ultimately leads to bacterial cell death. Some of the other critical extracted compounds in this study, namely phthalic acid, indole-3-acetic acid, and oleic acid, were also identified by previous researchers [46]. They screened for the anti-protozoal activity of supernatants containing secondary metabolites produced by *Photorhabdus* and *Xenorhabdus* species against antiprotozoal compounds using the easy PACId (promoter-activated compound identification) method [46]. Bacterial species in genera *Xenorhabdus* and *Photorhabdus* can produce various secondary metabolites to maintain their mutualistic symbiosis with the host EPNs. This study assessed all metabolites extracted functional compounds from the bacterial culture broth of *Xenorhabdus* and *Photorhabdus* to identify their virulent secondary metabolites against bacterial pathogens.

**Figure 8:**
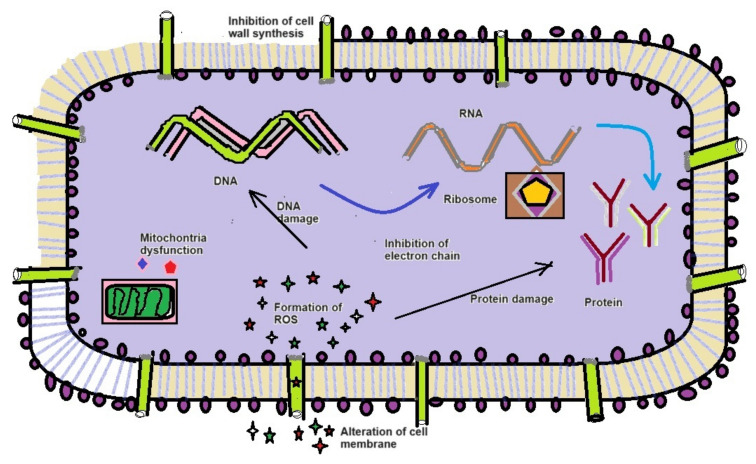
The general mechanisms of antimicrobial action on bioactive functional compounds from symbiotic bacteria.

Antimicrobial and growth inhibitory effects can be attributed to either the bacteria or their metabolites [47,48,49]. Our results are in line with those of previous studies and highlight the efficacy of extracts derived from these isolates against a spectrum of antibiotic-resistant bacteria [50]. Moreover, the antibacterial assay showed the inhibition of bacterial pathogens, namely *E. coli*, *S. aureus*, *B. subtilus*, *P. mirabilis*, *E. faecalis*, and *P. stutzeri*. Particularly, in the present study, *X. stockiae* strongly inhibited the growth of six strains of pathogenic bacteria compared to *P. luminescens*, followed by *E. coli*, *S. aureus*, and *E. faecalis*. This finding suggests that *X. stockiae* has the potential to inhibit bacterial pathogens. The general mechanisms of antibacterial activity highlight their interactions with nucleic acids, protein biosynthesis, protein-folding machinery, proteases, cell division machinery, cell wall biosynthesis, and the inhibition of lipopolysaccharide. By elucidating these diverse modes of action, the assessment underscores the versatility of bacterial secondary metabolites, thus acting as an effective antibacterial agent [51,52]. Figure 8 illustrates the mechanisms of antimicrobial action against pathogens using bacterial secondary metabolites based on a previous report [53]. For instance, the inhibition of the bacterial cell wall membrane occurs through the alteration of amino acid sugar in linear form, which cross-links through the peptidoglycan layer, as well as the inhibition of biochemical pathways associated with nucleic acid metabolism, translation, and transportation. On the other hand, changes in cell membrane integrity occur through an electrostatic interaction combined with a negatively charged membrane, leading to cell death. Other interesting aspects include (1) the inhibition of DNA synthesis via cross-linking DNA; (2) the prevention of DNA relaxation via the activation of DNA topoisomerase I; (3) disrupting the protein-folding cycle; and (4) proteolytic activity causing the degradation of DNA–RNA–protein replication, leading to cell damage.

Based on previous reports, we identified antimicrobial compounds from *X. stockiae* and *P. luminescens* that may inhibit bacterial growth [54]. This presents an opportune moment to explore the antimicrobial mechanism targeting specific bacterial cell membranes for novel drug discovery. For instance, *Xenorhabdus* has demonstrated the inhibition of closely related bacteria, while its metabolic compounds have shown efficacy against a spectrum of plant pathogens and some mammal disease pathogens [55,56,57,58]. One of the previous studies successfully utilized *X. stockiae* KT835471 extracellular metabolites as reducing and stabilizing agents for the formation of metal nanoparticles, and the results are invaluable in combating infectious diseases and addressing the growing challenge of antimicrobial resistance [59]. Additionally, the stupendous anticancer activity demonstrated by the secondary metabolites of symbiotic bacteria examined in human lung adenocarcinoma epithelial cells is particularly noteworthy. Similarly, *Photorhabdus* demonstrates broad-spectrum antimicrobial activity, inhibiting the growth of both bacteria and fungi. Previous studies have highlighted its effectiveness against bacterial pathogens such as *B. subtilis* and *E. coli*, *S. pyogenes*, as well as drug-resistant strains of *S. aureus*. Moreover, *Photorhabdus* exhibits inhibitory effects against a diverse range of fungi, including those responsible for plant diseases such as pecan scab [60]. Our investigation into the antibacterial inhibition effects of *P. luminescens* against pathogens, in agreement with the Orozco Group studies, revealed the wide range of bioactivity effects against various pathogens of secondary metabolites produced by *P. luminescens* sonorensis [61]. These compounds exhibit predominantly broad-spectrum antibacterial activity against a wide range of Gram-positive and Gram-negative bacteria that are of medical and agricultural significance. Moreover, the pathogenicity of *P. luminescens* toward insect larvae serves as a paradigm for understanding the symbiotic associations between bacteria and their hosts. The ability of *P. luminescens* to utilize siderophores for iron acquisition underscores the importance of this essential nutrient in the infection process. Iron is a critical cofactor for numerous cellular processes, and its sequestration by host organisms represents a common defense mechanism against invading pathogens [62]. By deploying siderophores, *P. luminescens* effectively competes with the host for iron resources, thereby ensuring its survival and proliferation within the host environment. On the other hand, researchers have highlighted the potential of utilizing bacterial associates as an eco-friendly alternative for controlling mosquito populations, particularly *Culex pipiens*, a significant vector of various infectious diseases [63]. By exploring the mosquitocidal activity of cell-free supernatants and cell suspensions of four different symbiotic bacteria, including *Xenorhabdus* and *Photorhabdus* spp., this study sheds light on a novel approach to mosquito control that could mitigate the adverse effects associated with synthetic insecticides. Consequently, we focused on studying novel antibacterial agents, predominantly bacterial products from various origins and geographic locations. Our investigations centered on identifying novel antibacterial agents from symbiotic bacteria (*Xenorhabdus* and *Photorhabdus).* Ideally, in the near future, their structures, biosynthesis, and mechanisms of action will be elucidated, and their production will be optimized.

## 4. Materials and Methods

### 4.1. Chemicals and Media

All the chemicals used for extraction and gas chromatography–mass spectrometry grade methanol was purchased from Naucalpan de Juárez, Mexico City, Merck, Mexico. Microbiological media were obtained from the Hi-Media Laboratory, Querétaro, México.

### 4.2. Identification, Extraction, and Molecular Characterization of Symbiotic Bacteria

Symbiotic bacteria (*Xenorhabdus* and *Photorhabdus*) were isolated in parallel from their nematode symbionts *Steinernema* and *Heterorhabditis*, respectively. For instance, symbiotic bacteria were extracted from newly emerged IJ nematodes, surface-sterilized, and then subjected to grinding. The final suspension was streaked on NBTA agar plates (0.004 nutrient agar, 2,3,5-triphenyltetrazolium chloride, and 0.025% bromothymol blue) according to a previous study [64]. The pure bacterial colonies were obtained from both nematodes. 

### 4.3. The 16S rDNA Sequencing and Phylogenetic Analysis

DNA extraction was carried out on the symbiont bacteria according to [65], with slight modifications. The small subunit (16s) rRNA was amplified with a PCR system, and the final products were separated by agarose gel and visualized using a transilluminator imaging system following a previous laboratory screening procedure [66]. Here, we used forward primer-(16s 20) 5′-AGA GTT TGA TCC TGG CTC-3′ and reverse primer-(16s 1390) 5′-GAC GGG CGG TGT GTA CAA-3′. The resultant high-quality sequences were analyzed with BLASTn (NCBI) to confirm the authenticity of the bacteria. 

### 4.4. Extraction of Bioactive Crude Compounds from Symbiotic Bacteria Using Ethyl Acetate

Bacterial colonies were established on NBTA agar plates. *Photorhabdus* and *Xenorhabdus* occur as two-phase variants (primary and secondary); yet, for the most part, only the primary phase produces antibiotics [67]. Thus, it was in our interest to maintain bacteria in the primary form. Soluble organic metabolites were then extracted from the bacterial cultures according to [68].

### 4.5. Purification of Bioactive Compound

Bacterial cultures were scaled up for metabolite isolation through liquid culture in TSY (tryptic soy broth +0.5% yeast extract). A loopful of bacteria was added to 50 mL of fresh TSY in a 300 mL Erlenmeyer flask and placed on a rotary incubator shaker at 25 °C and 130 rpm for 24 h. The cultures were then transferred to 900 mL TSY in 2 L flasks and placed on a rotary shaker at 25 °C for 96 h. Solvent extraction methods were utilized to separate bacterial metabolites based on their relative solubilities in two different immiscible liquids [69]. These steps were performed using separatory funnels and countercurrent distribution equipment. The supernatants were extracted three times with ethyl acetate, and organic fractions were dried with anhydrous ammonium sulfate, concentrated in a rotary evaporator, and dissolved in acetone. Furthermore, these two extracts were subjected to column chromatography packed with silica gel (230–400 mesh). The *X. stockiae* fractions were eluted with an increasing polarity gradient solvent system of acetone and methanol (100:0, 80:20, 60:40, 40:60, 20:80, and 0:100, *v*/*v*) to afford 8 fractions. Likewise, *P. luminescens* was eluted to generate 8 fractions. The active fraction was pooled and purified again using HPLC (Agilent 1220 infinity, Santa Clara, CA, USA) according to [70] with slight modifications. Each purified fraction was concentrated and checked for antibacterial activity against *E. coli*. After that, due to their higher inhibition rates, fraction 5 for *X. stockiae* and fraction 4 for *P. luminescens* were selected for screening.

### 4.6. Identification of Functional Bioactive Compounds by FTIR and GC–MS Analysis

The ethyl acetate extract was characterized by a Perkin Elmer spectrophotometer to determine the FTIR spectrum of the attached functional groups in a scanning range of 0 to 4000 cm^−1^ with a resolution of 4 cm^−1^. Gas chromatography–mass spectrophotometry was employed for the analysis of active constituents in *X. stockiae* and *P. luminescens*. Bacterial extracts using a GCMS-QP2010 Plus gas chromatograph (Shimadzu, Kyoto, Japan) were interfaced with a mass spectrometer. The sample was introduced into a glass injector working in split mode with helium as the carrier gas and a linear velocity pressure of 81.7 kPa. The following conditions were used: Rtx-5 MS fused silica capillary column (30 m × 0.25 mm. i.d. ×0.25 μm film thickness). The following temperatures were used: column oven temp.—80.0 °C, injection temp.—270.00 °C. The constituents were identified using commercial libraries.

### 4.7. Antibacterial Assay

Extracts of symbiotic bacterial (*X. stockiae* fraction 5 and *P. luminescens* fraction 4) active compounds were evaluated against both Gram-positive and Gram-negative bacteria. Bacterial strains *E. coli* (MTCC-2622), *S. aureus* (MTCC-96), *B. subtilus* (MTCC-2387), *P. mirabilis* (MTCC-1429), *E. faecalis* (MTCC-3159), and *Pseudomonas stutzeri* (MTCC-4831) were obtained from microbial-type culture collection and Gene Bank (https://mtccindia.res.in). They were subcultured in nutrient broth for 24 h at 30 °C. For biological activity, each strain was swabbed consistently into individual nutrient agar plates using sterile cotton swabs [71]. With a sterile micropipette, each extracted bacterial compound (*X. stockiae* fraction 5 was taken in concentrations of 25 μL, 50 μL, 75 μL, and 100 μL; *P. luminescens* fraction 4 was taken in concentrations of 25 μL, 50 μL, 75 μL, and 100 μL), was loaded into each well. Ampicillin solvent served as the positive control (10 μg/mL). After 3 min, sterilized paper disks were pressed lightly on the surface of pathogenic plates. The doses were selected based on preliminary data obtained from earlier studies. After 24 h incubation at 37 °C, the different levels of zone of inhibition (ZOI) were measured.

### 4.8. Statistics

The results are expressed as means and standard deviations in all experiments. All the experiments were conducted in at least triplicate.

## 5. Conclusions

In conclusion, the antimicrobial properties of natural products against *X. stockiae* and *P. luminesces* symbiotic bacteria present promising avenues for further exploration in the field of microbiology and biotechnology. Furthermore, the eco-friendly nature of natural products aligns with the growing demand for sustainable solutions in combating bacterial infections and antibiotic resistance. Despite the promising findings, it is important to acknowledge the challenges associated with translating these discoveries into practical applications. Further research is needed to elucidate the mechanisms of action underlying the antimicrobial activity of natural products against *Xenorhabdus* and *Photorhabdus* symbiotic bacteria, as well as their safety profiles and potential for resistance development. Additionally, the optimization of extraction methods, formulation techniques, and delivery systems will be crucial for maximizing the efficacy and stability of natural product-based antimicrobials. Overall, the exploration of natural products as antimicrobial agents against *Xenorhabdus and Photorhabdus* symbiotic bacteria offers exciting opportunities for innovation and development in the field of antimicrobial research. By harnessing the power of nature’s chemical diversity, we may uncover novel solutions to combat bacterial infections and contribute to the ongoing efforts to address the global challenge of antibiotic resistance.

## Figures and Tables

**Table 1:** Distribution and extraction of soil entomopathogenic nematodes and symbiotic bacteria using insect (*Galleria mellonella)* bait method from (agricultural and uncultured land) Querétaro, Mexico.

Sampling Site	Agriculture Crops/ Land	Recovered		Soil Type	Soil Temperature (°C)	Organic Content (%)	pH	Electrical Conductivity (mS/cm)	Symbiotic Bacteria Characteristics	
		*Steinernema* spp.	*Heterorhabditis* spp.						*Xenorhabdus* spp. (Amz 05)	*Photorhabdus* spp. (Amz 10)
Zone I (agricultural land)	Corn	++	-	sandy	29	4.9	7.25	0.93	Blue-color colony	Greenish yellow-color colony
	Rice	-	++	Loam clay	31	4.8	7.13	1.29	Bioluminescence (-) tive	Bioluminescence (+) tive
	wheat	++	-	loam	30	3.9	7.40	1.32	Catalase (-) tive	Catalase (+) tive
Zone II (uncultured land)	Forest	+	-	Silt	28	4.9	7.70	1.74	Absorption of bromothymol blue	Absorption of bromothymol blue
	Grasses	+	-	Silt	29	3.8	7.41	1.59	Growth on 28 °C	Growth on 28 °C
	Wasteland	++		Silt	30	4.1	7.23	1.03	Insect pathogenicity	Insect pathogenicity

++ shows two positive isolates; + shows one positive isolate; - Shows no isolate.

**Table 2:** FTIR spectrum peaks of *X. stockiae* crude compound in its possible assigned functional groups.

S. No.	FTIR Peaks (cm^−1^)	Possible Assigned Functional Groups
1	3443	Amine, N-H Stretch
2	1637	NH amide bend C-Br Bend
3	1100	ANAH and carbonyl (CAOA)
4	621	Carbonyl stretching (OC)

**Table 3:** FTIR spectrum peaks of *P. luminescens* crude compound in its possible assigned functional groups.

S. No.	FTIR Peaks (cm^−1^)	Possible Assigned Functional Groups
1	3436	Amine, N-H Stretch
2	2078	C≡N, Nitriles
3	1078	Carbonyl stretch
4	692	Alkyl Halides

**Table 4:** The eight potential HPLC fractions obtained from bacterial crude extracts of *X. stockiae*.

Fraction	Ret. Time (min)	Width (min)	Area (mAU*s)	Height (mAU)	Area%
1	9.393	0.3524	70.87936	3.20061	0.4908
2	10.122	0.3722	49.71435	1.83400	0.3443
3	14.197	0.0953	135.46420	19.44917	0.9380
4	17.607	0.1898	389.49295	26.14004	2.6971
5	18.755	1.2428	1.02867	114.86192	71.2308
6	23.703	0.4298	144.59134	4.28346	1.0012
7	28.894	0.5881	1209.27771	25.73598	8.3737
8	31.89	1.6023	2149.97168	15.93304	14.8876

**Table 5:** The eight potential HPLC fractions obtained from bacterial crude extracts of *P. luminescens*.

Fraction	Ret. Time (min)	Width (min)	Area (mAU*s)	Height (mAU)	Area%
1	10.637	0.2511	312.39853	16.05020	1.2641
2	11.795	0.5186	177.14165	4.21222	0.7168
3	17.842	0.0837	719.59143	120.40215	2.9119
4	18.842	1.2975	1.67736	182.29234	67.8757
5	23.503	0.2292	75.46230	4.55771	0.3054
6	23.715	0.2964	139.74609	6.30963	0.5655
7	28.887	0.8533	1907.49695	27.23028	7.7188
8	31.938	1.7550	4606.81006	31.15800	18.6418

**Table 6:** Summary of the fragments and retention times for significant compounds identified in the *X. stockiae* strain from ethyl acetate crude extract.

S. No.	R. Time	% of Area	Compound Name	Biological Activities	Reference
1	5.631	3.89	Nonanoic acid derivatives	Antimicrobial properties	[16]
2	5.823	4.27	Paromycin	Antibacterial agents	[17]
3	7.428	42.01	Pyrrolidinone	Bioactive compounds	[18]
4	8.848	3.98	Octodecanal derivatives	Bioactive compounds	[19]
5	13.198	4.53	Trioxa-5-aza-1-silabicyclo	Antimicrobial compounds	[20]
6	15.671	26.78	4-Octadecenal	Antifungal activity	[21]
7	22.981	2.84	Cyclopentanetridecanoic acid, Methyl ester	Antimicrobial peptide	[22]
8	23.978	4.629	Oleic Acid	Antifungal compound	[23]
9	31.98	1.876	1,2-benzenedicarboxylicacid	Bioactive molecules	[24]

**Table 7:** Summary of the fragments and retention times for significant compounds identified in the *P. luminescens* strain from ethyl acetate crude extract.

S. No	R. Time	% of Area	Compound Name	Biological Activities	Reference
1	6.328	2.95	Indole-3-acetic acid, methyl ester	Bioactive compound	[25]
2	7.810	59.43	Piperidinol derivatives	Antimicrobial agents	[26]
3	8.302	5.78	Phthalic acid	Bioactive compound	[27]
4	15.114	49.43	Nemorosonol	Bioactive metabolites	[28]
5	16.831	5.187	Octahydro-7-methyl-3-methylene	Insecticidal activity	[29]
6	20.302	3.651	1-eicosanol	Bioactive compound	[30]
7	25.220	2.284	Octadecanoic acid, Methyl ester	Antimicrobial activity	[31]
8	26.897	1.211	unsaturated fatty acids	Anti-inflammation	[32]

## Data Availability

Data are contained within the article and Supplementary Materials.

## Supplementary Materials

The following supporting information can be downloaded at: https://www.mdpi.com/article/10.3390/molecules29122914/s1, Figures S1–S10: Following some important functional bioactive compounds (ten) located in the crude extract on *X. stockiae* and *P. luminescens* using Gas chromatography-mass spectrometry with main constituents through NISIT Library [1,2- Benzenedicarboxylic acid, Acetic acid, Hydroxybenzoic acid, Octodecenol, Nonanoic acid, Octodecanic acid, Oleic acid methyl ester, Phthalic acid, Piperidenyl, Tetradecanic acid and Linoleic acid].
