# Supplementary material for: Preliminary Screening on Antibacterial Crude Secondary Metabolites Extracted from Bacterial Symbionts and Identification of Functional Bioactive Compounds by FTIR, HPLC and Gas Chromatography–Mass Spectrometry
Source: Molecules. 2024 Jun 19;29(12):2914. doi: 10.3390/molecules29122914 (PMC11206551; doi:10.3390/molecules29122914)
Supplement: Supplementary file 1 [file molecules-29-02914-s001.zip › molecules-3013414-supplementary.pdf]

Supplementary material: Following some important functional bioactive compounds (eleven) located in the crude extract on *X. stockiae* and *P. luminescens* using Gas chromatography-mass spectrometry with main constituents through NISIT Library [1,2- Benzenedicarboxylic acid, Acetic acid, Hydroxybenzoic acid, Octodecenol, Nonanoic acid, Octodecanic acid, Oleic acid methyl ester,Phthalic acid, Piperidenyl, Tetradecanic acid and Linoleic acid].

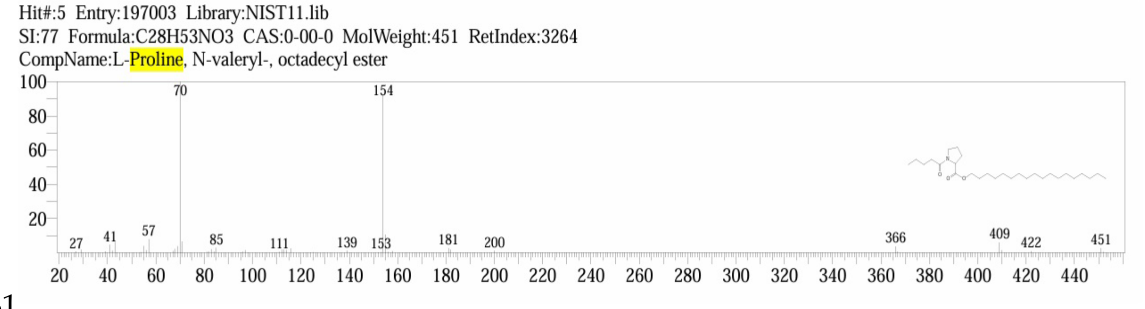

S1

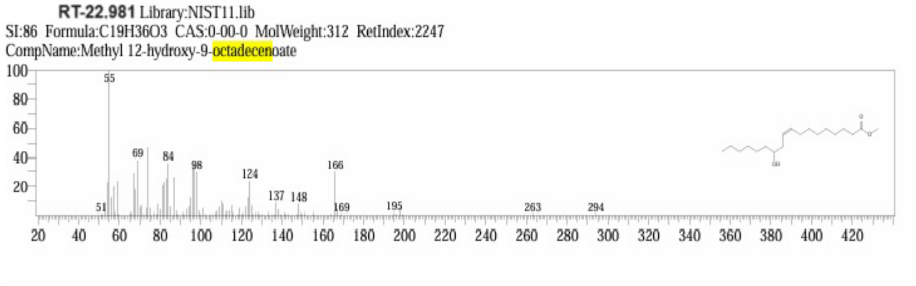

S 2

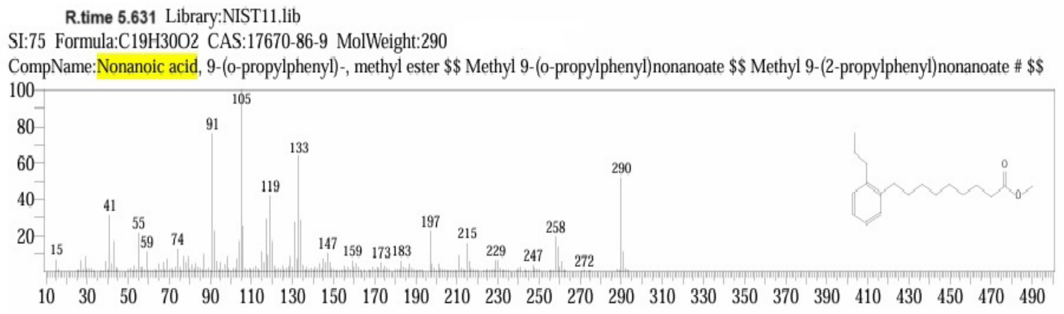

S3.

RT-8.848 Library:NIST11.lib
SI:96 Formula:C19H38O2 CAS:112-61-8 MolWeight:298 RetIndex:2077
CompName:Methyl stearate $$ Octadecanoic acid, methyl ester $$ Stearic acid, methyl ester $$ n-Octadecanoic acid, methyl ester $$ Kemester 9718 $$ Methyl n-octadecanoate $$ Methyl octadecanoate $$ Metholene 2218 $$ Emery 2218 $$ Kemester 9018 $$ Methyl ester of octadecanoic acid $$ Kemester 4516 $$ NSC 9418 $$

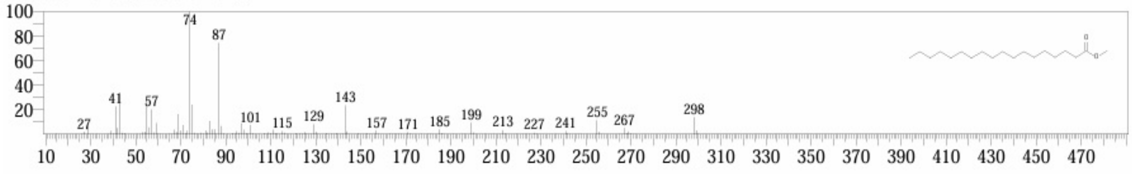

S4.

RT-23.978 Library:NIST11.lib
SI:87 Formula:C15H28O2 CAS:56219-06-8 MolWeight:240 RetIndex:1688
CompName:Methyl myristoleate $$ cis-9-Tetradecenoic acid, methyl ester $$ (Z)-9-Tetradecenoic acid, methyl ester $$ Methyl cis-9-tetradecenoate $$ Myristoleic acid methyl ester $$

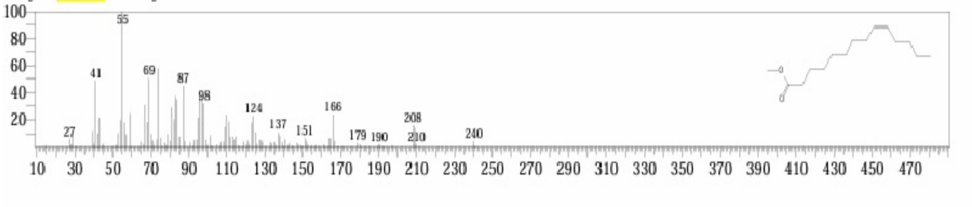

S5.

RT-8.302 Library:NIST11.lib
SI:66 Formula:C25H24O4 CAS:0-00-0 MolWeight:388 RetIndex:3062
CompName:Phthalic acid, 3,5-dimethylphenyl 4-isopropylphenyl ester

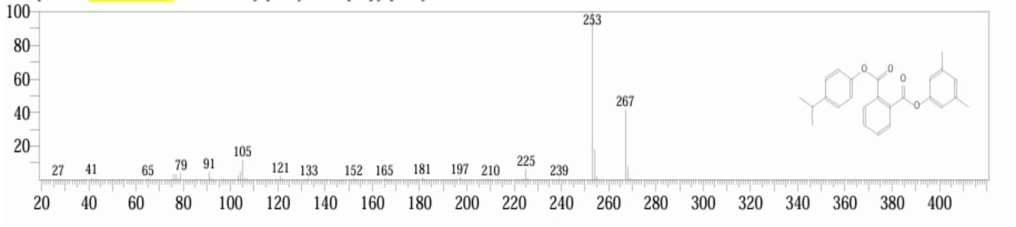

S6.

S6.

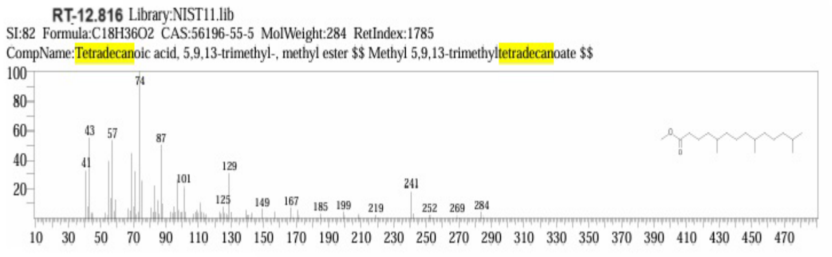
RT-12.816 Library:NIST11.lib
SI:82 Formula:C18H36O2 CAS:56196-55-5 MolWeight:284 RetIndex:1785
CompName:Tetradecanoic acid, 5,9,13-trimethyl-, methyl ester $$ Methyl 5,9,13-trimethyltetradecanoate $$

S7.

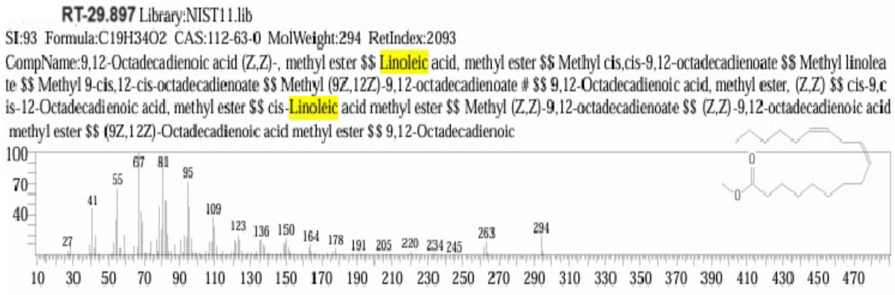
RT-29.897 Library:NIST11.lib
SI:93 Formula:C19H34O2 CAS:112-63-0 MolWeight:294 RetIndex:2093
CompName:9,12-Octadecadienoic acid (Z,Z)-, methyl ester $$ Linoleic acid, methyl ester $$ Methyl cis,cis-9,12-octadecadienoate $$ Methyl linoleate $$ Methyl 9-cis,12-cis-octadecadienoate $$ Methyl (9Z,12Z)-9,12-octadecadienoate # $$ 9,12-Octadecadienoic acid, methyl ester, (Z,Z) $$ cis-9,cis-12-Octadecadienoic acid, methyl ester $$ cis-Linoleic acid methyl ester $$ Methyl (Z,Z)-9,12-octadecadienoate $$ (Z,Z)-9,12-octadecadienoic acid methyl ester $$ (9Z,12Z)-Octadecadienoic acid methyl ester $$ 9,12-Octadecadienoic

S8.

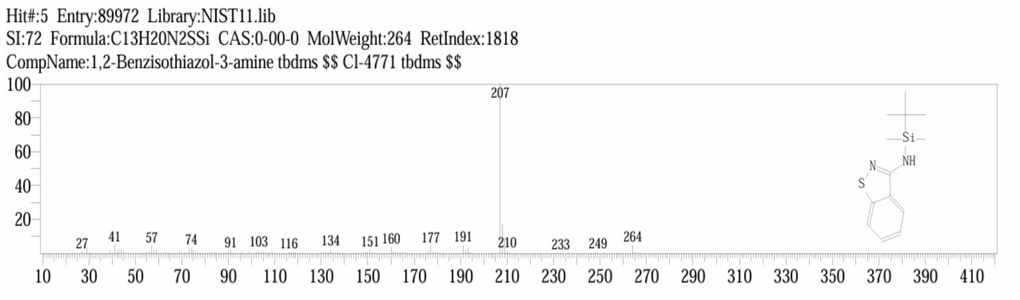
Hit#:5 Entry:89972 Library:NIST11.lib
SI:72 Formula:C13H20N2SSi CAS:0-00-0 MolWeight:264 RetIndex:1818
CompName:1,2-Benzisothiazol-3-amine tbdms $$ CI-4771 tbdms $$

S9

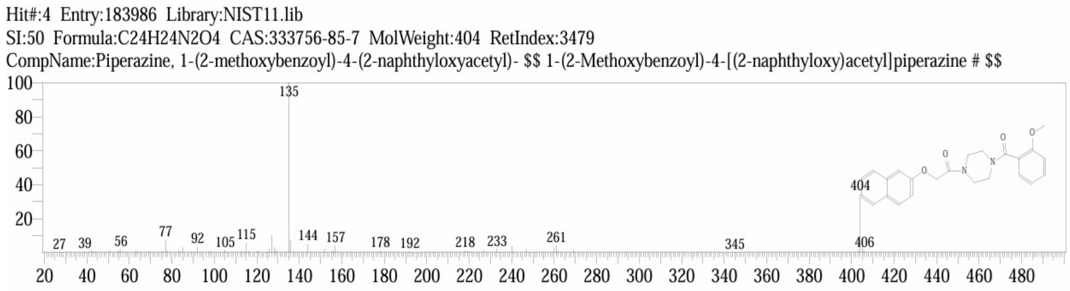
Hit#:4 Entry:183986 Library:NIST11.lib
SI:50 Formula:C24H24N2O4 CAS:333756-85-7 MolWeight:404 RetIndex:3479
CompName:Piperazine, 1-(2-methoxybenzoyl)-4-(2-naphthyloxyacetyl)- $$ 1-(2-Methoxybenzoyl)-4-[(2-naphthyloxy)acetyl]piperazine # $$

S10
